# Safety and efficacy of a low-dose combination of midazolam, alfentanil, and propofol for deep sedation of elderly patients undergoing ERCP

**DOI:** 10.1186/s12876-024-03197-9

**Published:** 2024-04-02

**Authors:** Yanping Zhang, Ning Zhang, Jing Hu, Changlin Liu, Guanjun Li

**Affiliations:** 1 Department of Anesthesiology, Cheeloo College of Medicine, Shandong Provincial Third Hospital, Shandong University, No.12 Wuyingshan Middle Road, Jinan, Shandong 250000 China; 2 Department of Cardiopulmonary rehabilitation, Cheeloo College of Medicine, Shandong Provincial Third Hospital, Shandong University, No.12 Wuyingshan Middle Road, Jinan, Shandong 250000 China

**Keywords:** Endoscopic retrograde cholangiopancreatography, Elderly, Deep sedation, Midazolam, Alfentanil, Propofol

## Abstract

**Background:**

Proper sedation of patients, particularly elderly individuals, who are more susceptible to sedation-related complications, is of significant importance in endoscopic retrograde cholangiopancreatography (ERCP). This study aims to assess the safety and efficacy of a low-dose combination of midazolam, alfentanil, and propofol for deep sedation in elderly patients undergoing ERCP, compared to a group of middle-aged patients.

**Methods:**

The medical records of 610 patients with common bile duct stones who underwent elective ERCP under deep sedation with a three-drug regimen, including midazolam, alfentanil, and propofol at Shandong Provincial Third Hospital from January 2023 to September 2023 were retrospectively reviewed in this study. Patients were categorized into three groups: middle-aged (50–64 years, *n* = 202), elderly (65–79 years, *n* = 216), and very elderly (≥ 80 years, *n* = 192). Intraoperative vital signs and complications were compared among these groups.

**Results:**

The three groups showed no significant difference in terms of intraoperative variation of systolic blood pressure (*P* = 0.291), diastolic blood pressure (*P* = 0.737), heart rate (*P* = 0.107), peripheral oxygen saturation (*P* = 0.188), bispectral index (*P* = 0.158), and the occurrence of sedation-related adverse events including hypotension (*P* = 0.170) and hypoxemia (*P* = 0.423).

**Conclusion:**

The results suggest that a low-dose three-drug regimen consisting of midazolam, alfentanil, and propofol seems safe and effective for deep sedation of elderly and very elderly patients undergoing ERCP procedures. However, further studies are required to verify these findings and clarify the benefits and risks of this method.

## Introduction

Endoscopic retrograde cholangiopancreatography (ERCP) is a diagnostic and therapeutic technique widely applied in various pancreatobiliary diseases [1]. Proper sedation of patients is of great significance in ERCP for patient comfort, convenience of physicians, reaching therapeutic goals, and preventing ERCP-related complications [2, 3].

Propofol is a non-opioid, non-barbituric anesthetic agent with short action and fast recovery and is widely utilized for anesthesia in a broad spectrum of surgeries as well as in patients undergoing ERCP [4]. Propofol alone is inadequate for semi-invasive procedures because it lacks analgesic action. Moreover, it can lead to the loss of airway reflexes, blood pressure drop, reduced cardiorespiratory function, and apnea at deep anesthetic levels [2, 3]. Combining propofol with an opioid is an effective approach to enhancing its sedative effects, avoiding its overuse, and consequently providing optimal sedation and analgesia without increased side effects [5, 6]. Alfentanil, as a potent opioid analgesic with rapid onset and short action time [7], has found broad application in recent years and is widely used as supplemental analgesia to anesthetic agents in various surgical procedures [8, 9]. Alfentanil combined with propofol has been utilized in ERCP procedures, showing low side effects and desirable safety [9]. Midazolam, a benzodiazepine with properties of rapid onset, short duration of action, and low toxicity [10], has proven to be a useful premedication to provide sedation, amnesia, and anxiolysis in a wide variety of procedures, including gastrointestinal endoscopy [11] and ERCP [2].

Midazolam combined with alfentanil and propofol has led to positive synergistic effects, enhancing the therapeutic action of each drug [7]. Additionally, it has been shown that their combination contributes to the use of smaller doses of individual drugs, thereby minimizing the sedation-related side effects [12, 13]. In a study including patients undergoing cardioversion with sedoanalgesia, the same three-drug combination was found to be associated with rapid onset, early recovery time, and less respiratory depression [12]. The results of a study on patients undergoing complicated oral surgeries with conscious sedation suggested that using these three drugs decreased total doses of each sedative agent and provided favorable hemodynamic stability and sedation quality in the patients [13]. The midazolam-alfentanil-propofol combination was also proved safe and efficient in providing appropriate sedation for patients undergoing colonoscopy [7, 14] and gastrointestinal endoscopy [15, 16].

With the aging of the global population [17] and the high prevalence of pancreatobiliary conditions in elderly patients [1, 2], the application of ERCP as a gold standard for treating biliary and pancreatic diseases [18] is ever-growing. On the other hand, due to the high prevalence of comorbidities in elderly patients, they are more prone to sedation-related complications, making safe sedation a significant concern in this group of patients [5, 6]. Ensuring safe and tolerable sedation while avoiding oversedation risks and adverse events is essential for improved management and better outcomes in elderly patients. Given that the combination of midazolam, alfentanil, and propofol, has shown significant clinical benefits for sedation management during various procedures as mentioned above [7, 12–16], using them in elderly patients undergoing ERCP may exhibit effective and safe outcomes. However, a paucity of studies have concentrated on elderly patients undergoing ERCP under deep sedation.

The present study evaluated the safety of a low-dose three-drug regimen consisting of midazolam, alfentanil, and propofol for deep sedation of elderly patients undergoing ERCP compared to a group of middle-aged patients.

## Methods

The medical records of patients who underwent ERCP procedures at Shandong Provincial Third Hospital (Shandong, China) from January 2023 to September 2023 were retrospectively reviewed in this study. The inclusion criteria were therapeutic ERCP for treating common bile duct stones, elective ERCP, age ≥ 50 years, and ERCP under deep sedation with propofol, alfentanil, and midazolam. Patients with the following conditions were excluded from the study: emergency ERCP; failed ERCP and need of surgical treatment for any reason, large stones that could not be removed from the common bile, duodenal diverticula, general anesthesia, and ASA status IV or more. Since malignancy is often associated with different underlying medical conditions that may affect the observed outcomes, the patients with malignancy were excluded from this study. A total of 610 patients met the study inclusion and exclusion criteria. The following data were collected for all patients: age, gender, weight, BMI, American Society of Anesthesiologists (ASA) classification, comorbidities, vital signs of patients including systolic blood pressure (SBP), diastolic blood pressure (DBP), heart rate (HR), peripheral oxygen saturation (SpO_2_) at different time points, bispectral index (BIS), procedure duration, dose of propofol, dose of alfentanil, time to awake, and sedation-related adverse events, such as hypotension, hypertension, hypoxemia, respiratory depression, bradycardia, tachycardia, and nausea and vomiting. Hypoxemia was defined as SpO2 less than 90% [18–20], while SpO2 below 90% that lasts more than the 10 s was considered respiratory depression [19]. Hypotension was defined as SBP below 90 mmHg [18, 19], and bradycardia was described as an HR of less than 50 beats per minute [19, 20]. Patients were categorized into three groups: middle-aged (50–64 years, *n* = 202), elderly (65–79 years, *n* = 216), and very elderly (≥ 80 years, *n* = 192). All the ERCP procedures were performed using a standard dudenoscope by four expert endoscopists with more than two years of experience conducting more than 250 ERCP procedures annually. The complexity level of ERCP procedures was graded as Class 2 and 3 according to the American Society for Gastrointestinal Endoscopy (ASGE) [21]. ERCP was performed in all patients under deep sedation based on ASA guidelines [22]. Sedation was administered by an anesthetist. Oxygen supplementation was provided by a nasal cannula at a rate of 5 L/min for all patients. Propofol, alfentanil and midazolam were sedative/analgesic drugs used for all patients. The BIS index was utilized to monitor the anesthesia depth of patients. BIS index is an objective and non-invasive monitoring approach for anesthesia and sedoanalgesia. It applies a sophisticated algorithm based on EEG parameters of the frontal cortex to achieve a quantitative score of anesthesia depth in the range of 0 (absence of brain activity) to 100 (fully awake) [19, 20]. The BIS index has been safely and effectively applied in different fields, including endoscopic procedures and ERCP, to guide titration of anesthetic agents, avoid excessive or inadequate anesthesia administration, and ensure adequate depth of anesthesia [15, 19, 20, 23, 24]. A BIS value of 50–60 indicates an appropriate level for deep sedation in the ERCP procedures [19, 25]. In order to induce and maintain deep sedation, all patients received intravenous administration of midazolam, alfentanil, and propofol. Induction was performed with midazolam 1 mg, alfentanil 5 µg/kg, and propofol 1–2 mg/kg, all three in single dose administration. Deep sedation was maintained with a continuous infusion of a mixture of propofol (3–6 mg/kg/h) and alfentanil (5–6 µg/kg/h) using a standard syringe pump. A level of deep sedation was targeted by adjusting the infusion rate of the mixture to keep BIS values between 50 and 60.

### Statistics

Statistical analysis was performed using SPSS statistical software version 25. Quantitative variables were expressed as mean ± standard deviation and categorical variables as frequency and percentage. A one-way ANOVA was utilized to compare quantitative variables, and a chi-square test was applied to compare categorical variables between the groups. Differences between the three groups regarding the rates of change in SBP, DBP, HR, SpO2, and BIS index were analyzed using repeated measure analysis of variance. A *p*-value less than 0.05 was considered statistically significant.

## Results

Overall, 610 patients who met the inclusion and exclusion criteria entered this study. Of these, 202 patients were in the middle-aged group (50–64 years), 216 belonged to the elderly group (65–79 years), and 192 were in the very elderly group (≥ 80 years). Table 1 provides the basic characteristics of patients by age group. It can be observed that the three groups significantly differed in terms of ASA class (*P* < 0.001), comorbidity (*P* < 0.001), and baseline values of SBP (*P* = 0.005), DBP (*P* < 0.001), HR (*P* < 0.001), SpO2 (*P* < 0.001), and BIS (*P* = 0.004).

**Table 1:** Basic characteristics of patients

Parameter	Age 50–64 *n* = 202	Age 65–79 *n* = 216	Age ≥ 80 *n* = 192	*P*-Value	
Age (y)	60.7 ± 2.7	73.5 ± 4.4	86.9 + 4.1	*P* < 0.001	
Gender					
Male	107 (53.0)	121 (56.0)	114 (59.4)	0.441	
Female	95 (47.0)	95 (44.0)	78 (40.6)		
Weight (kg)	62.9 ± 8.1	62.3 ± 5.0	61.5 ± 6.1	0.106	
BMI (kg/m^2^)	22.7 ± 2.7	22.6 ± 2.2	22.4 ± 2.5	0.408	
ASA					
I	113 (55.9)	24 (11.1)	0 (0.0)	*P* < 0.001	
II	71 (35.1)	160 (74.1)	114 (59.4)		
III	18 (8.9)	32 (14.8)	78 (40.6)		
Comorbidity					
Hypertension	42 (20.8)	77 (35.6)	95 (49.5)	*P* < 0.001	
Diabetes	17 (8.4)	30 (13.9)	43 (22.4)	*P* < 0.001	
Myocardial infarction	10 (5.0)	20 (9.3)	25 (13.0)	0.020	
Cerebral infarction	1 (0.5)	11 (5.1)	19 (9.9)	*P* < 0.001	
Renal disease	5 (2.5)	8 (3.7)	15 (7.8)	0.030	
Liver disease	4 (2.0)	9 (4.2)	12 (6.3)	0.102	
all	68 (33.7)	142 (65.7)	148 (77.1)	*P* < 0.001	
Baseline SBP	132.2 ± 13.6	133.0 ± 17.5	137.2 ± 17.9	0.005	
Baseline DBP	75.0 ± 6.8	75.9 ± 7.6	79.0 ± 7.2	*P* < 0.001	
Baseline HR	75.2 ± 7.1	73.1 ± 7.2	76.3 ± 5.4	*P* < 0.001	
Baseline SpO2	97.6 ± 1.1	97.7 ± 1.0	97.2 ± 1.2	*P* < 0.001	
Baseline BIS	92.9 ± 1.8	93.2 ± 1.7	92.6 ± 1.8	0.004	

Data are presented as mean ± standard deviation or number (percentage)

BMI Body mass index, ASA American society of anesthesiologists, SBP Systolic blood Pressure, DBP Diastolic blood pressure, HR Heart rate, SpO_2_ Saturation of Peripheral Oxygen, BIS Bispectral Index

**Fig. 1:**
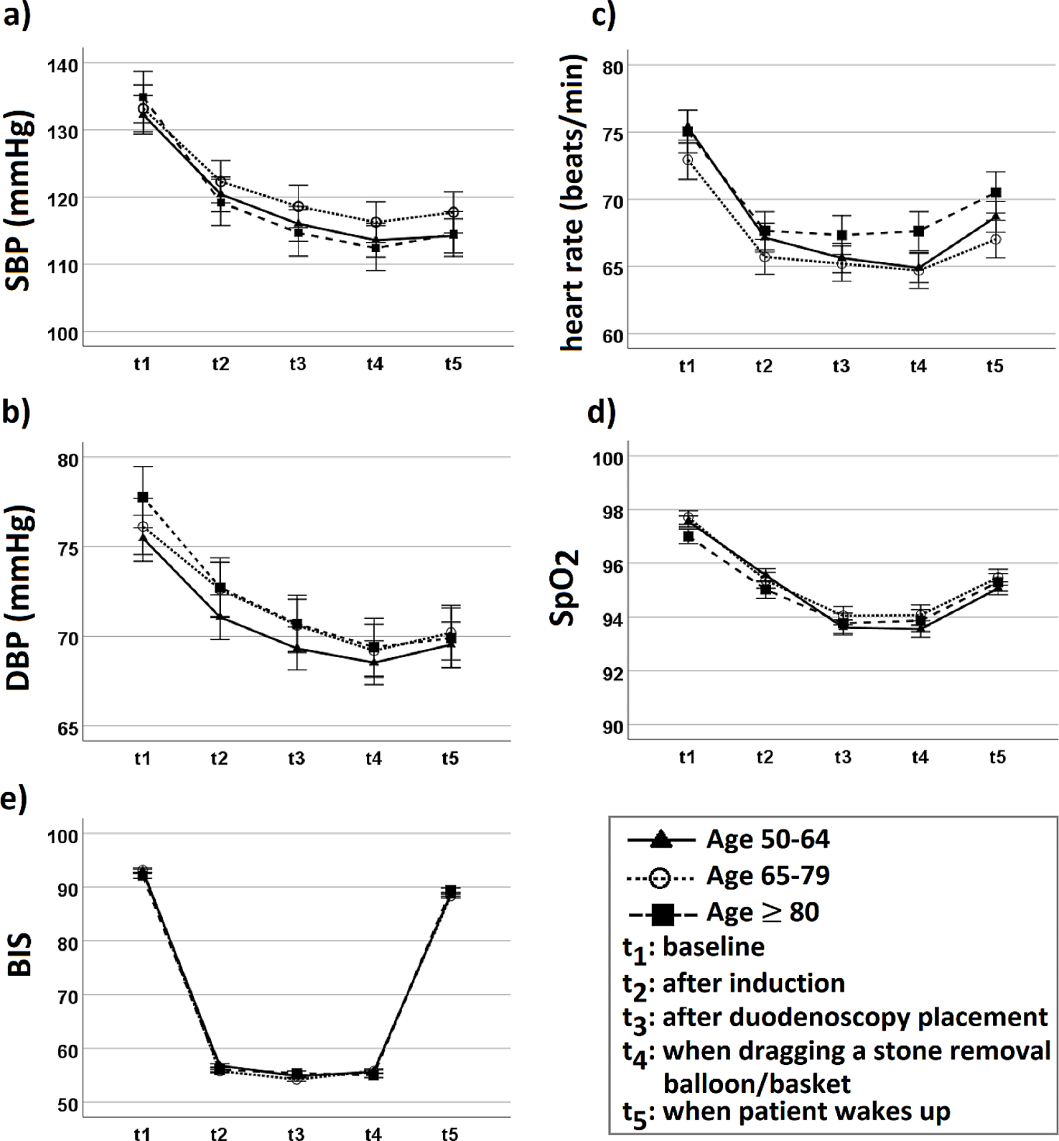
Variations in (**a**) systolic blood pressure, (**b**) diastolic blood pressure, (**c**) heart rate, (**d**) peripheral oxygen saturation, and (**e**) bispectral index in the middle-age, elderly, and very elderly patients

Figure 1 exhibits the variations in SBP, DBP, HR, SpO_2_, and BIS of the patients in the three age groups at various time points. Three groups showed no significant difference in variation of the above parameters at measured time points (*P* = 0.291, *P* = 0.737, *P* = 0.107, *P* = 0.188, and *P* = 0.158 for SBP, DBP, HR, SpO_2_, and BIS, respectively).

Table 2 presents the intraoperative data of the studied patients. The results showed a significant difference in the total doses of propofol and alfentanil used for middle-aged patients compared to elderly and very elderly patients (*P* < 0.001). However, no significant differences were noticed among the three groups in terms of procedure duration (*P* = 0.174), time to awake (*P* = 0.083), and occurrence of sedation-related adverse events including hypotension (*P* = 0.170) and hypoxemia (*P* = 0.423).

**Table 2:** Intraoperative data of patients

Parameter	Age 50–64 *n* = 202	Age 65–79 *n* = 216	Age ≥ 80 *n* = 192	*P*-Value
procedure duration (min)	66.4 ± 14.8	67.5 ± 9.3	68.8 ± 13.1	0.174
total dose of propofol (mg/kg)	5.6 ± 0.9	4.4 ± 0.4	4.3 ± 0.6	*P* < 0.001
total dose of alfentanil (µg/kg)	11.6 ± 1.5	10.6 ± 0.8	10.7 ± 1.1	*P* < 0.001
total dose of midazolam (mg)	1	1	1	-
Time to awake (min)	5.7 ± 2.4	5.3 ± 1.8	5.5 ± 1.5	0.083
Hypotension	9 (4.5)	4 (1.9)	10 (5.2)	0.170
Hypoxemia	7 (3.5)	5 (2.3)	9 (4.7)	0.423
Other complications *	0 (0.0)	0 (0.0)	0 (0.0)	-

Data are presented as mean ± standard deviation or number (percentage)

BMI Body mass index, ASA American society of anesthesiologists, SBP Systolic blood Pressure, DBP Diastolic blood pressure, HR Heart rate, SpO_2_ Saturation of Peripheral Oxygen, BIS Bispectral Index

*: hypertension, bradycardia, tachycardia, respiratory depression, nausea and vomiting

## Discussion

The present study evaluated the safety of deep sedation with a combination of midazolam, alfentanil, and propofol for ERCP in elderly patients compared to middle-aged patients. The results demonstrated that, overall, deep sedation of ERCP patients with the three-drug regimen was associated with few and minor side effects. Furthermore, very elderly and elderly patients showed no significant difference in the incidence of sedation-related complications compared to the middle-aged group.

Despite maintaining a deep sedation level in the patients under study, all cardiopulmonary complications were minor and non-serious. Hypotension occurred in 1.9–5.2% and hypoxemia in 2.3–4.7% of the patients, with no cases of hypertension, bradycardia, tachycardia, respiratory depression, nausea and vomiting, or mortality observed in patients. All patients developed transient hypoxemia, responded to jaw lifting, and did not require further interventions. Hypotension cases were managed with low-volume intravenous fluid or vasopressors and had no interference with the procedure. In general, a paucity of data is available on the efficacy and safety of the three-drug combination of midazolam, alfentanil, and propofol for sedation in various procedures and its sedation-related complications [7, 12–16]. The literature review revealed no information on elderly patients undergoing an ERCP procedure. However, our findings are comparable to those of Ho et al.’s study [7], where a combination of midazolam, alfentanil, and propofol was applied for deep sedation of patients undergoing diagnostic esophagogastroduodenal endoscopy and colonoscopy. In their study, 9.3% of patients developed hypotension, which was mild and transient in nature, and only 0.8% needed vasopressors. Furthermore, they reported hypoxemia in 3.1% of patients, all responding to the chin lift maneuver. Consistent with our study, no cases of bradycardia were observed. A recent study by Lin et al. [15] utilized a combination of midazolam, alfentanil, and propofol for moderate to deep sedation in patients candidate for advanced endoscopy and reported a much higher frequency of sedation-related complications compared to our study, including the need for nasal airway use (5.7%), hypotension requiring norepinephrine (3%), and bradycardia requiring atropine (1%). This inconsistency might be due to the difference in procedures, mostly endoscopic ultrasound in their study, and the inclusion of ASA class IV patients and those with malignancies. In another study by Arıcan et al. [14] using alfentanil-propofol-midazolam combination for sedation of colonoscopy candidate patients, hypoxemia (SpO2 < 90%) occurred in 5.4% of patients, all of which responded to chin lift. Hypotension was noted in 3.3% of patients, with none requiring vasopressors. No cases of bradycardia were reported, which agrees with our findings. In the study of Arıcan et al. [14], 2.2% of the patients also developed SpO_2_ < 85%, thereby requiring mask ventilation. However, no such cases were observed in our study. This difference may be attributed to the administration of a fixed dose of propofol (10–20 mg) for maintaining sedation in all patients in their study. In Ozkan et al.‘s study [12] on patients undergoing electrical cardioversion, the incidence rate of hypoxemia was 9.1% in patients under sedation with midazolam-propofol-alfentanil, which was higher than in our study. However, all hypoxemia cases in their study were transient and responded to tactile or vocal stimulus without the need for assisted ventilation. The frequency of hypotension in their study was 6.1%. No incidence of bradycardia was reported, making it comparable to our findings. In a study conducted by Offord et al. [13] on patients undergoing complex oral surgery procedures under conscious sedation, no sedation-related complications were noted in patients receiving the midazolam-propofol-alfentanil combination, which is attributable to the lighter sedation levels in their study.

Our results indicated that while elderly and very elderly patients had higher ASA class and a greater frequency of comorbidities, their sedation-related complications with the three-drug regimen were generally low and were similar to those in middle-aged patients. There was no significant difference in the occurrence of hypoxemia and hypotension between the three groups and no cases of respiratory depression, bradycardia, nausea and vomiting, or mortality were observed in any of the age groups. As far as we know, limited studies are available regarding the safety of deep sedation in elderly patients undergoing ERCP, each with a different sedation regimen. For instance, Amornyotin et al. [2] suggested that the combination of midazolam, fentanyl, and propofol is safe and effective for deep sedation in elderly patients undergoing ERCP. In their study, the frequency of hypoxemia was 1.8-2%, lower than in our study, while the frequency of hypotension was 18.3–24.5%, higher than our findings. In addition, upper airway obstruction and bradycardia occurred in 2-3.7% and 1.9% of their studied patients, respectively. The main reason for these discrepancies may be different anesthesia protocols and the inclusion of ASA IV patients in their study. Nevertheless, they noted that serious adverse events in elderly patients were rare, and all complications were manageable. The results of Chen et al.‘s study [26] revealed that a combination of dexmedetomidine and propofol for deep sedation in elderly patients candidate for ERCP can reduce sedation-related side effects and provide better hemodynamic conditions compared to propofol alone. Their reported rate of adverse events, including hypoxemia (4.2–36%), hypotension (16.7–60%), bradycardia (12-58.3%), and hypertension (16-20.8%), was higher than that of our study with different anesthesia protocols being possibly a key factor in this discrepancy. However, adverse events in their study were mostly non-serious, transient, or well-managed with early interventions and no cases of arrhythmia, cardiac arrest, or death were noticed in their patients. A study by Tokmak et al. [27] on patients undergoing ERCP under deep sedation with a combination of midazolam, ketamine, and pethidine found that sedation-related complications in very elderly patients (above 80 years old) were not significantly different from those in middle-aged patients (under 65 years old). When compared to our study, their results showed a lower incidence of hypoxia (3.1%) and a higher incidence of bradycardia (2%), tachycardia (32%), and hypertension (36%). This discrepancy may primarily be due to differences in administered anesthetic-sedative agents. Although significant differences among studies preclude their direct comparison, when compared to previous studies, the three-drug regimen, including midazolam, alfentanil, and propofol, evaluated in this study seems to provide adequate safety for deep sedation during ERCP in elderly patients.

Multiple factors may have contributed to the low sedation-related complications in elderly patients in this study. Despite the higher frequency of comorbidities and ASA class in elderly patients compared to middle-aged ones, the general health conditions of the elderly patients were relatively acceptable, and none were assigned to ASA class ≥ 4, which justifies their lower baseline vulnerability to anesthesia-related complications. The administration of low-dose midazolam may reduce the initial required dose of propofol, thereby lowering the risk of propofol-related cardiopulmonary complications [28]. Besides, as previously mentioned, the combination of midazolam, alfentanil, and propofol has shown positive synergistic effects [7], which may allow to achieve the desired sedation depth at lower doses of each drug [12, 13] and potentially reduce the risk of sedation-related adverse events. Oxygen supplementation for all patients at a rate of 5 L/min via a nasal cannula throughout the procedure, as recommended in other studies as well [7], might be involved in reduced incidence of hypoxemia.

Additionally, drug titration using BIS monitoring is likely to play a role in decreasing the sedation-related adverse events in the current study. In their study on patients undergoing ERCP under deep sedation, Kilic et al. [24] suggested that BIS monitoring shows a high agreement with the Ramsay Sedation Score (RSS), which is a conventional clinical method for sedation depth evaluation and can be used to predict early respiratory depression during ERCP sedation. Inal et al. [20] found that compared to using the Ramsay Sedation Score, titrating propofol based on BIS monitoring during sedation for ERCP resulted in a considerable decrease in the number of drops in SpO_2_ below 90%. They concluded that BIS monitoring can be beneficial in lowering the risk of respiratory depression during sedation. Another study by Lin et al. [15] on patients undergoing advanced gastrointestinal endoscopy under moderate-to-deep sedation found that titration of the depth of sedation by BS monitoring, when compared to Modified Observer’s Assessment of Alertness/Sedation Scale, can significantly decrease mean propofol infusion rate while considerably improving the satisfaction level of endoscopists. Paspatis et al. [19] revealed that using the BIS index as a primary target to adjust the level of sedation in patients candidate for ERCP under deep sedation led to a substantial reduction in propofol consumption dose. A systematic review and meta-analysis of 11 randomized controlled trials, including 1039 patients undergoing endoscopic procedures (sedation in 526 patients with BIS monitoring and in 513 patients with standard monitoring) indicated that BIS monitoring was associated with significantly lower total propofol consumption compared to standard monitoring [23]. Aujla et al. [29] compared sedation safety data in ERCP patients before and after the introduction of BIS monitoring. According to their findings, BIS monitoring was associated with a significant reduction in sedation-related adverse events, significantly reduced need for reversal agents, and improved recovery time. By allowing precise titration of propofol and alfentanil, BIS-guided sedation seems to prevent excessive sedation, help maintain vital signs within a safe range, and reduce sedation-related adverse events, as seen in the present study. However, considerable controversy exists regarding the accuracy and clinical value of the BIS index for sedation in endoscopic procedures [15]. Some studies have failed to show benefits for BIS monitoring in terms of reduced doses of anesthetic agents or sedation-related adverse events [30, 31]. Further studies are needed to clarify the BIS benefits in sedation titration for elderly patients.

### Limitations

A major limitation of this study was its retrospective design. Additionally, this study was a single-center study with a relatively limited sample size. Another limitation was that we only examined the patients with ASA class less than IV undergoing elective procedures and excluded those with malignancies. These limitations can introduce selection bias in our study and compromise the generalizability of our results. Thus, our findings should be interpreted cautiously, and further studies are required in this area.

## Conclusion

Altogether, our findings suggested that a low-dose three-drug regimen, including midazolam, alfentanil, and propofol, seems safe and effective for deep sedation of elderly and very elderly patients undergoing ERCP procedures. However, given the limitations of the study, caution should be exercised when interpreting and generalizing these findings. Further studies, primarily randomized controlled trials, are required to test and verify these findings and clarify the potential benefits and risks of this method.

## Data Availability

The datasets used and/or analysed during the current study are available from the corresponding author on reasonable request.
